# Ethical issues in epidemiologic research and public health practice

**DOI:** 10.1186/1742-7622-3-16

**Published:** 2006-10-03

**Authors:** Steven S Coughlin

**Affiliations:** 1 Epidemiology and Applied Research Branch, Division of Cancer Prevention and Control, National Center for Chronic Disease Prevention and Health Promotion, Centers for Disease Control and Prevention, 4770 Buford Hwy, NE (K-55), Atlanta, GA 30341

## Abstract

A rich and growing body of literature has emerged on ethics in epidemiologic research and public health practice. Recent articles have included conceptual frameworks of public health ethics and overviews of historical developments in the field. Several important topics in public health ethics have also been highlighted. Attention to ethical issues can facilitate the effective planning, implementation, and growth of a variety of public health programs and research activities. Public health ethics is consistent with the prevention orientation of public health. Ethical concerns can be anticipated or identified early and effectively addressed through careful analysis and consultation.

## Introduction

A rich and growing body of literature has emerged on ethics in epidemiologic research and public health practice [1-11]. Recent articles have included conceptual frameworks of public health ethics and overviews of historical developments in the field [7,8,11]. Several important topics in public health ethics have also been highlighted [7,11,12].

This article provides an overview of ethical issues in epidemiologic research and public health practice for readers who do not necessarily have an in-depth knowledge of public health ethics. In the discussion that follows, a summary is provided of current definitions and conceptualizations of public health ethics and key ethical concerns in the field.

## Definitions and conceptualizations of public health ethics

The starting point for conceptualizations of public health ethics has often been general definitions of public health, such as the definition provided by the Institute of Medicine in 1988: "Public health is what we, as a society, do collectively to assure the conditions in which people can be healthy." As noted by Childress et al. [8], "*Public health is primarily concerned with the health of the entire population, rather than the health of individuals. Its features include an emphasis on the promotion of health and the prevention of disease and disability; the collection and use of epidemiological data, population surveillance, and other forms of empirical quantitative assessment; a recognition of the multidimensional nature of the determinants of health; and a focus on the complex interactions of many factors – biological, behavioral, social, and environmental – in developing effective interventions*." Public health activities also include community collaborations and partnerships for health and the identification of priorities for public health action.

Previous authors have identified ethical issues and core values in public health, and highlighted differences and similarities between public health ethics and other areas of bioethics [5,7]. Public health ethics, which can be defined as the identification, analysis, and resolution of ethical problems arising in public health practice and research, has different domains from those of medical ethics. Ethical concerns in public health often relate to the dual obligations of public health professionals to acquire and apply scientific knowledge aimed at restoring and protecting the public's health while respecting individual autonomy [1,3]. Ethics in public health involves an interplay between protecting the welfare of the individual, as in medicine, and the public health goal of protecting the public welfare [1]. Other ethical concerns in public health relate to the need to ensure a just distribution of public health resources [13]. Public health ethics has a broad scope that includes ethical and social issues arising in health promotion and disease prevention, epidemiologic research, and public health practice [5,7].

In conceptualizing public health ethics and distinguishing it from other areas of bioethics, previous authors have often highlighted mandatory or coercive public health measures that are authorized by public health law (for example, quarantining people with contagious diseases) or activities that may infringe upon personal privacy or autonomy, such as public health surveillance. In many public health activities there is a tension between concerns over personal liberties and individual autonomy and public health perspectives, which may be utilitarian, paternalistic, or communitarian. Communitarian perspectives may favor limiting individual autonomy for the sake of the common good or public interest [7].

Despite the importance of mandatory public health activities required by law, many examples of voluntary public health activities can be cited. Public health surveys, for instance, depend upon the support and informed consent of members of the public. In deliberating about ethical questions in their own public health activities, public health professionals have increasingly referred to explications of moral reasoning methods useful for public health research and practice.

## Moral reasoning in public health

Moral reasoning involves deliberating about ethical questions and reaching a decision with the help of judgment and rational analysis. In such deliberations, particular decisions and actions may be justified by ethical theory or an integrated body of rules and principles. Two theories have commonly been cited in public health research and practice: deontological and utilitarian [14]. Deontological theories (sometimes referred to as Kantian theories) hold that people should not be treated as means to an end and that some actions are right or wrong regardless of the consequences. Deontological theories provide strong support for protecting research participants and whole communities of people, even if protections for human subjects slow research or the acquisition of knowledge.

Utilitarian theories, on the other hand, strive to maximize beneficial consequences. The principle of utility requires aggregate or collective benefits to be maximized. From a utilitarian perspective, the principle of utility is the ultimate ethical principle from which all other principles are derived [14]. Utilitarian theories provide strong justification for public health programs such as mandatory vaccination programs for children and the fluoridation of public water supplies.

Different methods of moral reasoning have been applied to ethical decision making in public health research and practice [5]. Two approaches have figured most prominently: the principle-based approach to moral reasoning explicated by Beauchamp and Childress, and case-based methods such as casuistry [15].

### Principle-based approaches

Principle-based approaches to moral reasoning were developed to address ethical issues in clinical medicine and are not necessarily the optimal approach for analyzing ethical issues in public health. The four principles of beneficence, nonmaleficence, justice, and respect for autonomy are mentioned in ethics guidelines drafted for public health professionals, although the guidelines do not provide an exhaustive account of how the principles can be used as a framework for ethical decision making [9,16]. Principles such as justice also figure prominently in still-evolving ethics frameworks that have been proposed for public health [8,11,13].

The principles of beneficence, nonmaleficence, autonomy, and justice, as explained by Beauchamp and Childress [17], seek to reduce morality to its basic elements and to provide a useful framework for ethical analysis in the health professions. The principles do not provide a full philosophical justification for decision making, however. In situations where there is conflict between principles, it may be necessary to choose between them or to assign greater weight to one. Practical problems in public health ethics require that these principles be made more applicable through a process of specification and reform [14]. Ongoing progressive specification is needed as new issues and concerns arise.

The ethical principle of beneficence requires that potential benefits to individuals and to society be maximized and that potential harms be minimized [17]. Beneficence involves both the protection of individual welfare and the promotion of the common welfare. This principle underlies ethical rules and norms that require that public health institutions act in a timely manner on the information they have and that they expeditiously make the information available to the public [9]. The principle of nonmaleficence requires that harmful acts be avoided. However, the principle of nonmaleficence does not preclude balancing potential harms against potential benefits [14]. The principle of autonomy focuses on the right of self-determination. Respect for the individual is a principle rooted in the Western tradition, which grants importance to individual freedom in political life, and to personal development.

Principles of justice are also important [11,13,14]. Utilitarian theories of justice emphasize a mixture of criteria so that public utility is maximized. From this perspective, a just distribution of benefits from public health programs or research is determined by the utility to all affected. As noted by Childress et al. [8], public health activities are generally understood to be *consequentialist *in that the primary end that is sought is the health of the public. An egalitarian theory of justice holds that each person should share equally in the distribution of the potential benefits of health care resources such as screening services. Other theories of justice hold that society has an obligation to correct inequalities in the distribution of resources, and that those who are least well off should benefit most from resources such as screening services. Such theories of justice provide considerable support for maximizing benefits to medically underserved people [13,18].

### Case-based approaches to moral reasoning

While many general ethical questions have been answered on the basis of general principles and theories, the specific decisions that emerge in particular cases may remain unaddressed by the principles. Such decisions are often made by focusing on the circumstances of the case at hand and the moral context in which the case rests. Case-based methods such as casuistry are grounded in analogical reasoning, appeal to paradigmatic cases, and practical judgment [5,14].

In casuistry, which in contemporary bioethics has been championed by Albert Jonsen and Stephen Toulmin [15,19], decision making takes place at the level of the particulars of the case itself. Given a case and a particular decision to be made, a casuist need not refer directly to a particular theory. Rather, maxims are identified that have bearing on the case. Maxims are wise, pithy, rule-like sayings such as "tell the truth" or "be compassionate."

Casuistry requires a clear exposition of the facts that surround a case. A decision must then be made about which maxim is the most appropriate to "rule" or govern the case. Different circumstances or facts might call for a different maxim. A claim or judgment is then made regarding the case. The claim is backed by a form of logical reasoning described in terms of grounds or relevant circumstances, maxims, and the backings or more general notions that support the maxims. The descriptions of the case, including the circumstances, maxims, and logical thought, constitute its basic structure or morphology [14]. Placing a particular case alongside other similar cases has been referred to as taxonomy.

Casuistic reasoning begins with relatively clear, paradigmatic cases in which some ethical norm indicates the right course of action. Judgment is necessary to determine which norm applies in a complicated or ambiguous case.

### Other approaches to moral reasoning

Other approaches to moral reasoning, such as rights-based theories, duty-based theories, contractarianism, the ethics of care, narrative ethics, and communitarianism have not been widely applied in public health. Virtue ethics and the moral rule-based system of Gert and Clouser, however, have been discussed as potential alternatives to other leading approaches to moral reasoning in public health ethics [5,20].

Moral disagreements can sometimes be resolved by obtaining further facts about matters at the center of the controversy or by more clearly defining the language used by the disputing parties [14]. Other steps that can be taken to resolve moral controversies include using examples and counter-examples and analyzing arguments to expose their inadequacies, gaps, and fallacies. In addition, moral problems can sometimes be resolved by getting the disputing parties to adopt a new policy or code, such as ethics guidelines for epidemiologists [14].

## Ethical issues in epidemiology and public health practice

The results of epidemiologic research studies contribute to generalizable knowledge by elucidating the causes of disease; by combining epidemiologic data with information from other disciplines such as genetics and microbiology; by evaluating the consistency of epidemiologic data with etiological hypotheses; and by providing the basis for developing and evaluating health promotion and prevention procedures [21]. The primary professional roles of epidemiology are the design and conduct of scientific research and the public health application of scientific knowledge. This includes reporting research results and maintaining and promoting health in communities. In carrying out these professional roles, epidemiologists often encounter a number of ethical issues and concerns that require careful consideration. Many of these issues have been addressed in the literature on ethics in epidemiology and public health practice including ethics guidelines.

## Issues dealt with in ethics guidelines for epidemiologists and the published literature

Ethical and professional norms in epidemiology have been clarified in ethics guidelines for epidemiologists and other public health professionals [16,22-24]. Ethics guidelines such as those developed for the Industrial Epidemiology Forum, the International Society for Environmental Epidemiology, and the American College of Epidemiology provide useful accounts of epidemiologists' obligations to research participants, society, employers, and colleagues. Ethics guidelines for environmental epidemiologists drafted by Colin Soskolne and Andrew Light, which were adopted by the International Society for Environmental Epidemiology in 1999, highlight the important obligations that epidemiologists have to communities that are affected by environmental hazards [22]. The ethics guidelines adopted by the American College of Epidemiology discuss core values, duties, and virtues in epidemiology; the professional role of epidemiologists; minimizing risks and protecting the welfare of research participants; providing benefits; ensuring an equitable distribution of risks and benefits; protecting confidentiality and privacy; obtaining informed consent; submitting proposed studies for ethical review; maintaining public trust; avoiding conflicts of interest and partiality; communicating ethical requirements; confronting unacceptable conduct; and obligations to communities [16]. International guidelines for ethical review of epidemiologic studies were published by the Council of International Organizations of Medical Sciences [24]. The CIOMS guidelines draw a distinction between epidemiologic research and routine practice (for example, outbreak investigations and public health surveillance) and consider some of the issues associated with obtaining informed consent in epidemiologic studies. Specific ethical issues arising in epidemiologic research and public health practice that have been highlighted in ethics guidelines include minimizing risks and providing benefits, informed consent, avoiding and disclosing conflicts of interest, obligations to communities, and the institutional review board system.

### Minimizing risks and providing benefits

Ethical concerns in epidemiology and public health practice often relate to the obligations of health professionals to acquire and apply scientific knowledge aimed at maintaining and restoring public health while respecting individual rights. Potential societal benefits must often be balanced with risks and potential harms to individuals and communities, such as the potential for stigmatization or invasions of privacy.

Epidemiologists have ethical and professional obligations to maximize the potential benefits of studies to research participants and to society, and to minimize potential harms and risks. In addition, these obligations are often legal or regulatory requirements, such as U.S. federal regulations protecting human research participants (45 CFR 46). The risks of epidemiologic studies and practice activities can be minimized by rigorously protecting the confidentiality of health information, as discussed below. Although the risks posed by epidemiologic studies are often minor compared with those that may be associated with clinical trials and other experimental studies, participants in epidemiologic studies may be burdened by a loss of privacy, by time spent completing interviews and examinations, and by possible adverse psychological effects such as enhanced grief or anxiety [25]. Such risks and potential harms can be minimized by careful attention to study procedures and questionnaire design, for example, by limiting the length of interviews or by scheduling them on a date that is less likely to result in adverse psychological effects.

Minimizing risks and potential harms and maximizing potential benefits are particularly important in epidemiologic studies of vulnerable populations. Examples include studies of children, prisoners, some elderly people, and populations that are marginalized or socioeconomically disadvantaged.

A further obligation is the need to ensure that the burdens and potential benefits of epidemiologic studies are distributed equitably. The potential benefits of epidemiologic research are often societal in nature, such as obtaining new information about the causes of diseases, or identifying health disparities across groups defined by race, ethnicity, socioeconomic status, or other factors [25]. Research participants may receive direct benefits from participation in some studies, such as when a previously unrecognized disease or risk factor is detected during examinations. The balance of risks and potential benefits of epidemiologic studies are considered not only by individual researchers but also by members of human subjects committees such as institutional review boards in the United States.

### Avoiding and disclosing conflicts of interest

Other ethical issues that arise in the professional practice of epidemiology relate to how best to deal with potential conflicts of interest, in order to maintain public trust in epidemiology and sustain public support for health research. Recent media reports about previously undisclosed conflicts of interest in the United States and other countries have raised public awareness of the potential for conflicts of interest in clinical research and epidemiology, and about the need for institutions and individual researchers to address such conflicts. Conflicts of interest can affect scientific judgment and harm scientific objectivity. Studies have suggested that financial interests and researchers' commitment to a hypothesis can influence reported research results [26]. To address such concerns, funding agencies and research institutions have taken steps such as adopting new training programs that encourage researchers to avoid or disclose conflicts of interest, and revising or strengthening institutional rules and guidelines. Professional societies and medical associations have also issued policy statements and recommendations about how best to address conflicts of interest in clinical research [27]. Researchers should disclose financial interests and sources of funding when publishing research results. It may also be important to disclose information about potential or actual financial conflicts of interest when obtaining informed consent from research participants. A related issue is that health researchers should avoid entering into contractual agreements that prevent them from publishing results in a timely manner [16]. Communicating research results in a timely manner, without censorship or interference from the funder, is essential for maintaining public trust [9].

### Obligations to communities

The obligations of epidemiologists to study participants have been highlighted in several reports [16,22]. These obligations include communicating the results of epidemiologic studies at the earliest possible time, after appropriate scientific peer review, so that the widest possible audience stands to benefit from the information. Epidemiologists should strive to carry out studies in a way that is scientifically valid and interpret and report the results of their studies in a way that is scientifically accurate and appropriate. In addition, epidemiologists should respect cultural diversity in carrying out studies and in communicating with members of affected communities. Other obligations to community members and to research participants have been highlighted in ethics guidelines for epidemiologists and public health institutions [9].

### Informed consent

Informed consent provisions in public health studies ensure that research participants make a free choice and also give institutions the legal authorization to proceed with the research [28]. Investigators must disclose information that potential participants use to decide whether to consent to the study. This includes the purpose of the research, the scientific procedures, anticipated risks and benefits, any inconveniences or discomfort, and the participant's right to refuse participation or to withdraw from the research at any time [45 Code of Federal Regulations (CFR) 46]. Informed consent requirements may be waived in exceptional circumstances when obtaining consent is impractical, the risks are minimal, and the risks and potential benefits of the research have been carefully considered by an independent review committee. For example, in some epidemiology studies involving the analysis of large databases of routinely collected information (for example, insurance claims data), it may not be feasible to recontact patients to ask them for their informed consent. Risks and potential harms in such studies may be very low, and risks may be further reduced by omitting personal identifiers from the computer databases.

Special considerations for obtaining informed consent may arise in public health studies of socioeconomically deprived people. People who have limited access to health care may misunderstand an invitation to participate in a study as an opportunity to receive medical care. In addition, they may be reluctant to refuse participation when the researcher is viewed as someone in a position of authority, such as a physician or university professor. Socioeconomically deprived people may also be more motivated to participate in studies involving financial incentives for participation. A further issue is that there is often a need to translate informed consent statements into a language other than English. The important issues that arise in international research conducted by researchers from countries such as the United States and Great Britain in developing countries have also received considerable attention [24,29].

### Privacy and confidentiality

One important way in which public health researchers reduce potential harms and risks to participants in epidemiologic studies is by rigorously protecting the confidentiality of their health information. Specific measures taken by researchers to protect the confidentiality of health information include keeping records under lock and key, limiting access to confidential records, discarding personal identifiers from data collection forms and computer files whenever feasible, and training staff in the importance of privacy and confidentiality protection [25]. Other measures that have been employed to safeguard health information include encrypting computer databases, limiting geographic detail, and suppressing cells in tabulated data where the number of cases in the cell is small [30].

In the United States, the Health Insurance Portability and Accountability Act (HIPAA) of 1996 took effect early in 2004 after extensive planning and discussion [31]. The new regulations provide protection for the privacy of certain individually identifiable health data, referred to as protected health information. The privacy rules permit disclosures without individual authorization to public health authorities authorized by law to collect or receive the information for the purpose of preventing or controlling disease, injury, or disability, including public health practice activities such as surveillance.

### The institutional review board system

The purpose of research ethics committees or institutional review boards (IRBs) is to ensure that studies involving human research participants are designed to conform with relevant ethical standards and that the rights and welfare of participants are protected. Human-subjects review by such committees ensures that studies have a favorable balance of potential benefits and risks, that participants are selected equitably, and that procedures for obtaining informed consent are adequate. In the United States, federal regulations to protect human research subjects (45 CFR 46) have resulted in a complex IRB system. Similar safeguards exist in many other countries.

Despite the important role played by research ethics committees and IRBs, researchers have sometimes expressed concern about the obstacles that human-subjects review can create. In some countries, human-subjects review has been streamlined with the use of standardized forms and review processes or by centralizing review by research ethics committees [32]. As previously mentioned, one of the important issues considered by research ethics committees and by individual researchers is the adequacy of provisions for obtaining the informed consent of study participants.

These are just some of the ethical issues addressed in ethics guidelines developed for epidemiologists and other public health professionals. Other issues addressed in the guidelines include those pertaining to scientific misconduct, intellectual property and data sharing, publication of research findings, and cross-cultural or international health research.

## Ethical issues in public health practice

An expanding body of literature has considered the important ethical issues that arise in such areas of public health practice as surveillance, emergency responses, and program evaluation [1,4,33-35]. In further specifying ethical norms in particular contexts, it is important to draw distinctions between epidemiologic research and public health practice activities. For example, requirements for submitting research protocols to an IRB do not necessarily apply to outbreak investigations and other emergency responses [36].

### Definitions of surveillance, emergency responses, and program evaluation

Surveillance can be defined as the ongoing, systematic collection, analysis, and interpretation of outcome-specific data, with the timely dissemination of these data to those responsible for preventing and controlling disease or injury [37]. A fundamental public health activity is to measure and monitor changes in health status, risk factors, and health service access and utilization. The effective dissemination of information is as important as data collection and analysis; the collected information must have a demonstrated utility [38].

Emergency responses and outbreak investigations can be defined as public health activities undertaken in an urgent or emergency situation, usually because of an imminent health threat to the population [39]. Sometimes this is because the public or government authorities perceive an imminent threat that demands immediate action. The primary purpose of the activity is to determine the nature and magnitude of a public health problem in the community and to implement appropriate measures to address the problem [39].

Field epidemiology and investigations of disease outbreaks require us to consider when the data are sufficient to take action rather than to ask what additional questions might be answered by the data [40]. The guidelines and approaches for conducting epidemiologic field investigations reflect the urgency of discovering causative factors and the need to make practical recommendations, such as during the SARS epidemic [41]. Program evaluation, on the other hand, refers to the systematic application of scientific and statistical procedures for measuring program conceptualization, design, implementation, and utility; the comparison of these measurements; and the use of the resulting information to optimize program outcomes [36,42,43].

Federal regulations (Title 45 CFR Part 46), which deal with issues such as IRB review and informed consent requirements, mostly address biomedical research [36,42,43]. These regulations define research as a systematic investigation, including development, testing, and evaluation designed to develop or contribute to generalizable knowledge. Although some public health activities can clearly be classified as either research or non-research activities for regulatory purposes, for other activities the classification is more difficult. For example, scientific knowledge generated in controlling a disease outbreak may turn out to be useful in other settings, even though generating generalizable knowledge was not the primary intent of the investigation [36].

In applying the federal regulations for protecting participants in public health research, U.S. agencies have distinguished health research and non-research public health practice activities. Research and non-research activities cannot be easily defined by the methods that are employed. For example, questionnaire development, laboratory analysis, and logistic regression techniques are commonly employed in etiologic studies with a case-control design, as well as in many case-control studies conducted as part of outbreak investigations. To address this issue, guidelines from the Centers for Disease Control and Prevention state that the major difference between research and non-research lies in the primary intent of the activity. The primary intent of research is to generate or contribute to generalizable knowledge. The primary intent of non-research activities in public health practice is to prevent disease or injury, improve health, and ensure the efficient and effective use of resources.

For example, surveillance projects are likely to be non-research when they involve the regular, ongoing collection and analysis of health-related data, conducted to monitor the frequency and distribution of diseases and health conditions in the population. Surveillance projects may have a research component when they involve the collection and analysis of health-related data conducted either to generate knowledge that is applicable to other populations and settings or to contribute to general knowledge about the health condition. Most emergency responses and outbreak investigations tend to be non-research because these projects are undertaken to solve an immediate health problem and any knowledge gained will likely benefit only the study participants or target population [36].

Although some ethical requirements, such as IRB review, do not apply equally to epidemiologic research and non-research public health practice activities, there are many important similarities between the ethics of epidemiologic research and non-research (for example, requirements for confidentiality protection in research and non-research disease surveillance systems). Investigators should carefully consider ethical issues in each project, regardless of whether it is research or public health practice.

### Ethical issues in public health surveillance

Ethics guidelines for public health surveillance have been developed for disease registry personnel, and a growing body of literature has evolved in this area, indicating increasing interest [4,33-35,44]. These developments are partly a response to public concern over the privacy and confidentiality of health information and technological advances such as the use of the Internet to disseminate data from surveillance systems and disease registries.

Data collected through surveillance systems provide for the ongoing evaluation of disease risk factors, incidence, and mortality, and allow for the evaluation of health care utilization, treatment, and disease prevention and control activities [45]. These and other benefits of public health surveillance must be balanced against possible risks and harms, such as infringements on personal privacy. The need to balance potential benefits against risks underlines the rule that surveillance data should not be collected if they will not be used [44]. Thus, public health professionals have ethical obligations to both maximize the potential benefits of routinely collected surveillance and disease registry data and minimize risks and potential harms. Steps taken to assure the quality of data collected by public health surveillance systems and disease registries maximize the potential benefits of the data. Registry data must be accurate, complete, and timely.

Potential harms and risks from the collection and use of surveillance and registry data include loss of privacy and harms resulting from breaches of confidentiality. These risks are remote possibilities because of the steps taken by public health professionals to safeguard the confidentiality of personally identifiable records in surveillance systems and registries, such as data encryption, written policies and procedures for confidentiality and disclosure of data, and training of staff.

The privacy rules included in HIPAA permit disclosures without individual authorization to public health authorities who can legally collect or receive the information for the purpose of preventing or controlling disease, injury, or disability. This includes public health practice activities such as surveillance.

## Health promotion and disease prevention

The potential benefits of disease prevention and health promotion efforts include a healthier society and reduced fiscal expenditure and increased productivity and efficiency [46]. Individual members of society can also benefit. There is a need to balance health as a value with values of privacy and autonomy (for example, in relation to immunization policies). Several authors have considered the circumstances under which personal autonomy can be abridged to promote the health of the whole community and the moral justification for coercive public health interventions and lifestyle strategies [47,48]. As noted by Lappe [1], "*From an ethical perspective, the extent to which [compulsive public health] interventions are justified depends on... the anticipated extent and kind of public benefit; the degree to which individual rights are restricted to achieve that benefit; and the ultimate distribution of both benefits and harms attendant to participation*."

In general, there is a need for voluntariness in health education, health promotion, and public health communication programs. The risks and potential harms of public health interventions include ineffective, counterproductive, or harmful interventions; unanticipated consequences; and labeling or stigmatizing of individuals [49]. Undue stress upon the individual's role in the cause of illness could lead to a "blame the victim" mentality [48]. The dilemma is how to advise people that they might be at risk for potentially serious health complications without labeling them, contributing to their anxiety, or adversely affecting their well-being [49].

Ethical considerations for prevention trials and community interventions include an assessment of risks and benefits, the need for voluntary participation and avoidance of excessive incentives, and justice-related issues. There is a need for sensitivity to ethnic and cultural habits and norms and to avoid "top-down" planning, in which the health concerns and self-defined information needs of the target population are ignored in favor of professional preoccupations and concerns. Such concerns have been successfully addressed through community-based participatory research, which is a collaborative, empowering process that helps develop competencies in communities [50]. Ethical issues in health communication include the need to avoid conflicts of interest, to present facts about health hazards or health opportunities in a truthful, balanced, and timely fashion, and to avoid distorting the facts or concealing ambiguities in the scientific evidence [49].

### Ethical issues in screening

Ethical issues also arise in public health screening programs [51]. Screening is the presumptive identification of an unrecognized disease or condition by the use of tests, examinations, or other procedures that can help identify a disease or disease precursor in apparently well people. People with positive or suspicious findings then undergo further evaluation or treatment. The ultimate objective of screening is to reduce the morbidity or mortality from a disease among the people screened.

Several frameworks for analyzing and addressing ethical and policy issues in public health screening programs have been proposed. In 1968, Wilson and Jungner [52] proposed 10 principles for mass screening programs. These principles are often cited in planning and evaluating population screening programs; they relate to the adequacy of the scientific evidence, the balance of risks and benefits, the availability of an effective treatment, the acceptability of the screening test to the population, and the costs and resources required [51]. Refinements have been proposed over the years, with further specification of the principles of screening [53-56]. Criteria for the effectiveness of clinical preventive services have been developed by the Canadian Task Force on the Periodic Health Examination [57] and by the U.S. Preventive Services Task Force [58]. Screening raises a number of important ethical issues around informed consent, privacy and confidentiality, risks and potential benefits, and the allocation of finite public resources for screening.

The principle of respect for individuals' freedom supports the right of participants to informed consent prior to screening [51]. Provisions for informed consent ensure that people undergoing screening make free choices, and encourage providers to act responsibly in their interactions with patients. Subjects should be given information about the procedure, the meaning of a positive or negative test result, and any appreciable risks or potential harms and benefits before undergoing screening [51]. To give informed consent for screening, participants need to understand the risk of a false-positive test result and the procedures that may follow it [59].

Principles of informed consent for screening have some features in common with emerging models of informed decision making and shared decision making for screening and other health care services [60]. Such models emphasize that people should be provided with balanced and relevant information so they can make informed decisions about screening options [61-63]. As discussed by Briss et al. [62], informed decision making occurs when the participant understands the nature of the disease or condition being addressed; understands the clinical service and its likely consequences, including risks, limitations, benefits, alternatives, and uncertainties; has considered his or her preferences as appropriate; has participated in decision making at a personally desirable level; and either makes a decision consistent with his or her preferences and values or elects to defer a decision to a later time.

Although public health screening is generally voluntary, some examples of mandatory screening can be cited. For example, most states require that infants be screened for certain genetic disorders, such as phenylketonuria (PKU). Infants are subject to the screening program unless their parents refuse for religious or philosophical reasons [51]. Public health officials may justify mandatory newborn screening programs, even without parental consent, under utilitarian principles authorizing state governments to protect children [51].

The potential benefits of screening include the early detection of disease and the prevention of serious illness or disability and improved survival. The societal benefits of screening include substantial reductions in morbidity and mortality [58]. Screening is undertaken for conditions that are important public health problems and those for which early detection and treatment are effective. If early treatment is not effective, then early detection alone merely extends the length of time the disease is known to exist, without extending survival [59]. Public health policy makers rely on information from randomized controlled trials and other sources to evaluate the effectiveness, potential benefits, and risks or potential harms of screening.

The potential harms and risks associated with screening also have to be taken into account, especially since screening programs are aimed at large numbers of apparently healthy people. Minor complications or infrequent adverse effects that would be acceptable in the treatment of a severe illness take on greater importance when screening asymptomatic people and require careful evaluation to determine whether the potential benefits exceed risks [58]. There may be risks associated with false-positive or false-negative test results. The potential harms of screening may also include "labeling" effects and the psychologic impact of test results or a diagnosis. If prognosis is not improved by presymptomatic detection, screening for a disease can cause anxiety without providing any benefit [56]. Medical information collected as part of screening should be rigorously safeguarded to protect patient privacy and confidentiality and to minimize risks or potential harms such as stigma or discrimination. Only a few specific exceptions exist, such as mandatory partner notification laws for HIV infection that physicians are legally required to follow in some states [64].

## Summary and conclusion

The burgeoning interest in ethical issues in epidemiologic research and public health practice reflects both the important societal role of public health and the growing public interest in the scientific integrity of health information and the equitable distribution of health care resources. Attention to ethical issues can facilitate the effective planning, implementation, and growth of a variety of public health programs and research activities. Seen from this perspective, public health ethics is consistent with the prevention orientation of public health. Ethical concerns can be anticipated or identified early and effectively addressed through careful analysis and consultation.
